# CRISPR/Cas13d-mediated efficient *KDM5B* mRNA knockdown in porcine somatic cells and parthenogenetic embryos

**DOI:** 10.1530/REP-21-0053

**Published:** 2021-06-07

**Authors:** Dengfeng Bi, Jing Yao, Yu Wang, Guosong Qin, Yunting Zhang, Yanfang Wang, Jianguo Zhao

**Affiliations:** 1 School of Life Sciences, University of Science and Technology of China, Hefei, China; 2 State Key Laboratory of Stem Cell and Reproductive Biology, Institute of Zoology, Chinese Academy of Sciences, Beijing, China; 3 Institute for Stem Cell and Regeneration, Chinese Academy of Sciences, Beijing, China; 4 Beijing Institute for Stem Cell and Regenerative Medicine, Beijing, China; 5 University of Chinese Academy of Sciences, Beijing, China; 6 Laboratory of Animal (Poultry) Genetics Breeding and Reproduction, Ministry of Agriculture, Institute of Animal Science, Chinese Academy of Agricultural Sciences, Beijing, China

## Abstract

An efficient mRNA knockdown strategy is needed to explore gene function in cells and embryos, especially to understand the process of maternal mRNA decay during early embryo development. Cas13, a novel RNA-targeting CRISPR effector protein, could bind and cleave complementary single-strand RNA, which has been employed for mRNA knockdown in mouse and human cells and RNA-virus interference in plants. Cas13 has not yet been reported to be used in pigs. In the current study, we explored the feasibility of CRISPR/Cas13d-mediated endogenous RNA knockdown in pigs. KDM5B, a histone demethylase of H3K4me3, was downregulated at the transcriptional level by 50% with CRISPR/Cas13d in porcine fibroblast cells. Knockdown of *KDM5B-*induced H3K4me3 expression and decreased the abundance of H3K27me3, H3K9me3, H3K4ac, H4K8ac, and H4K12ac. These changes affected cell proliferation and cell cycle. Furthermore, stable integration of the CRISPR/Cas13d system into the porcine genome resulted in the continuous expression of Cas13d and persistent knockdown of *KDM5B*. Finally, the RNA-targeting potential of Cas13d was further validated in porcine parthenogenetic embryos. By microinjection of Cas13d mRNA and gRNA targeting *KDM5B* into porcine oocytes, the expression of *KDM5B* was downregulated, the abundance of H3K4me3 increased as expected, and the expression of embryonic development-related genes was changed accordingly. These results indicate that CRISPR/Cas13d provides an easily programmable platform for spatiotemporal transcriptional manipulation in pigs.

## Introduction

The clustered regularly interspaced short palindromic repeats (CRISPR)/CRISPR-associated genes (Cas) system has been rapidly used for various genomic engineering in a wide range of organisms, from yeast to mammals (Cong *et al.* 2013, Hsu *et al.* 2014). Recently, a novel RNA-targeting CRISPR effector protein, called Cas13 (Koonin *et al.* 2017, Shmakov *et al.* 2017), has been shown to bind and cleave RNA rather than DNA substrates, suggesting a potential for RNA editing and programming.

**Figure 1:**
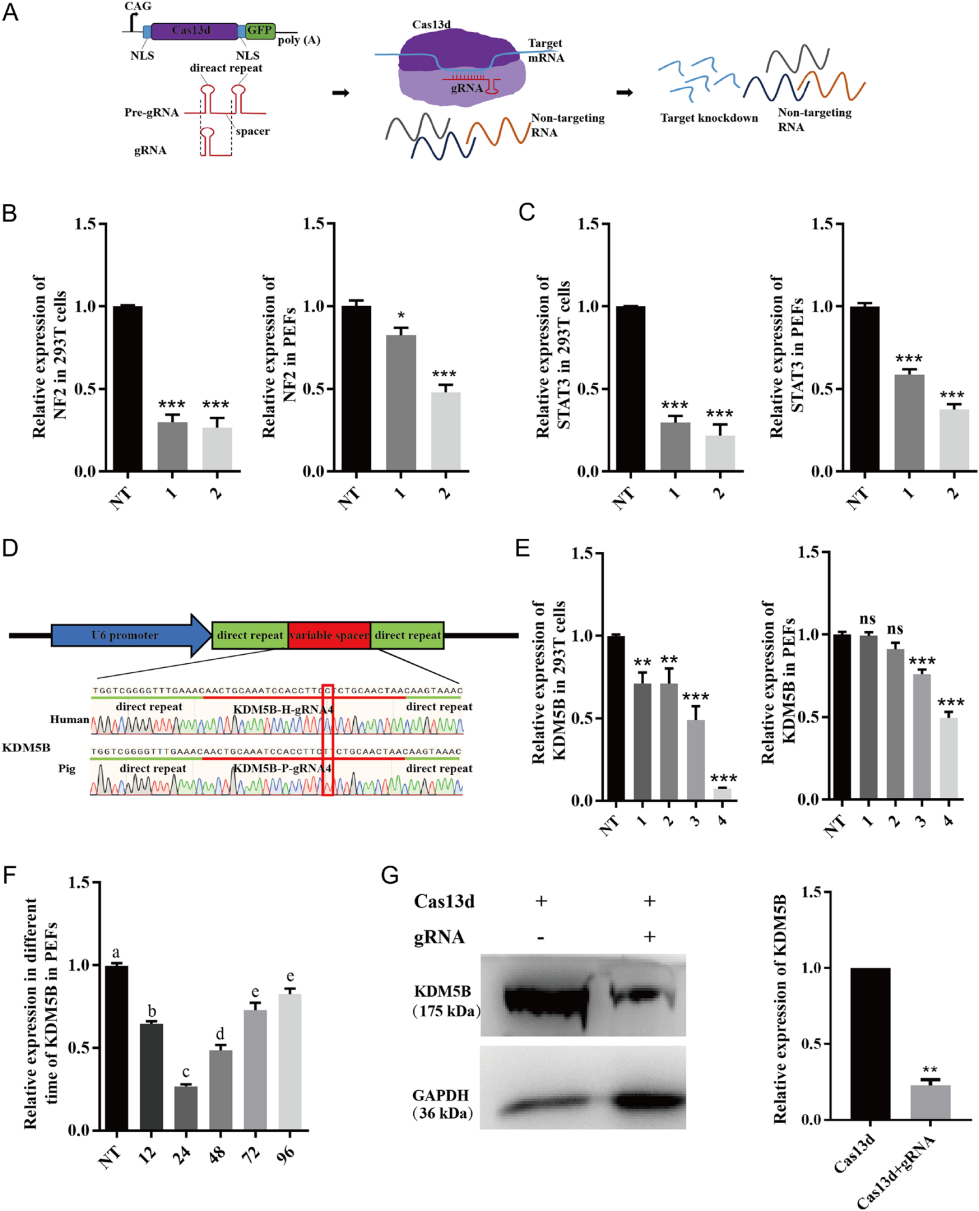
Cas13d-mediated RNA knockdown in human cells and pig cells. (A) Schematic of constructs encoding Cas13d and gRNA. NLS, nuclear localization signal. Pre-gRNA, unprocessed guide RNA containing a single 30nt spacer sequence flanked by two 36nt direct repeats. gRNA, matured guide RNA with 22nt spacer sequence. The purple box, Cas13d protein. (B and C) Cas13d-mediated knockdown of NF2 (B) and STAT3 (C) in 293T and PEF cells (left and right) as determined by quantitative PCR. 1 and 2, gRNA1 and gRNA2; NT, non-targeting gRNA. The transcript levels were normalized against GAPDH. Data are presented as mean ± s.e.m., *n*  = 3. *P < 0.05, **P < 0.01, ***P < 0.001. (D) Sequence of gRNA4 targeting human and pig KDM5B sequence. There is one base difference in gRNA4 targeting the human and pig KDM5B sequences, as indicated in red rectangle. (E) The knockdown of KDM5B mediated by Cas13d via transient transfection in 293T (left) and PEF cells (right) with different gRNAs (1–4) and non-targeting gRNA (NT). The transcript levels were normalized against GAPDH. Data are presented as mean ± s.e.m., *n*  = 3. *P < 0.05, **P < 0.01, ***P < 0.001. (F) Cas13d-mediated knockdown of KDM5B with gRNA4 at different time points post-transfection. The transcript levels were normalized against GAPDH. Data are presented as mean ± s.e.m., *n*  = 3. Values with different letter are significantly different, *P* < 0.05. (G) Western blot analysis of KDM5B expression. The quantitative result is shown on the right. Data are presented as mean ± s.e.m., *n*  = 3. **P < 0.01.

Cas13 proteins include four different subfamilies (Cas13a, Cas13b, Cas13c, and Cas13d), and all possess two Higher Eukaryotes and Prokaryotes Nucleotide-binding (HEPN) ribonuclease motifs (Shmakov *et al.* 2015, Tambe *et al.* 2018). After binding a precursor CRISPR-RNA (pre-crRNA), Cas13 cleaves the pre-crRNA array within the crRNA direct repeat to form mature Cas13-crRNA complexes. This HEPN domain can be converted to active conformation and cleaves target RNA molecules (Fig. 1A) (cis-cleavage), further cleaving random other RNA it encounters (trans- or collateral cleavage). CRISPR/Cas13d, a subtype of Cas13, similar to other members of the Cas13 family, comprises two parts: the programmable single-effector RNA-guided RNase Cas13 and a 64-66nt CRISPR-RNA which recognizes a 24nt sequence on the target RNA. Comparing with the Cas13a and Cas13b effectors, Cas13d shows advantages in its significantly greater transcript knockdown efficiency, smaller protein size, and no protospacer flanking sequence (PFS) preference (Konermann *et al.* 2018, Yan *et al.* 2018). Recently, CRISPR-Cas13d has been developed for unique applications, such as interference against RNA viruses in plants (Mahas *et al.* 2019), knockdown of DNA ligase IV in mouse cells to promote homologous recombination (HDR) efficiency (Pan *et al.* 2019), and downregulation of Ptbp1 gene to converted glia to neurons in mice (Zhou *et al.* 2020), which suggests great prospects of CRISPR/Cas13d in gene regulation, gene therapy, and anti-virus applications. Further, a recent study showed that the RNA editing activity of Cas13d from *Ruminococcus flavefaciens* performed better than RNAi and dCas9-mediated CRISPR interference (CRISPRi) across three endogenous transcripts tested, exhibiting an average knockdown efficiency of 96% compared to 65% for RNAi and 53% for CRISPRi (Granados-Riveron & Aquino-Jarquin 2018, Konermann *et al.* 2018). In addition, no significant off-target transcriptional perturbations were observed with Cas13d, while widespread off-targeting changes were detected with RNAi (Granados-Riveron & Aquino-Jarquin 2018, Konermann *et al.* 2018).

Histone methylation is one of the most important epigenetic regulations that play a critical role in mammalian embryonic development. KDM5B, an H3K4me3 histone demethylase, is implicated in cancer development (Catchpole *et al.* 2011, Xhabija & Kidder 2019) and proliferation (Catchpole *et al.* 2011) and is also indispensable for embryonic stem cell (ESC) self-renewal, cell fate, and murine embryonic development (Kidder *et al.* 2014). We previously showed that maternal *KDM5B* is essential to porcine preimplantation embryo development (Huang *et al.* 2015). Knockdown of *KDM5B* by morpholino antisense oligonucleotides (MOs) disturbed the balance of bivalent H3K4me3-H3K27me3 modification at the blastocyst stages (Huang *et al.* 2015, Dahl *et al.* 2016), which indicated that *KDM5B* is critical to chromatin remodeling during porcine preimplantation embryo development.

Although CRISPR/Cas13d was reported to be highly efficient for RNA knockdown in mouse and human cells (Konermann *et al.* 2018), whether it can be used to interfere with endogenous genes in pigs remains unknown. In the current study, we first demonstrate the feasibility of CRISPR/Cas13d in mediating endogenous RNA knockdown in porcine fibroblast cells and early embryos by direct microinjection, which indicates CRISPR/Cas13d as a flexible platform for studying RNA in pig cells and early embryo development.

## Materials and methods

### Plasmids

The expression plasmid of Cas13d (#109049, Addgene, Watertown, MA, USA) and pre-gRNA (#109054, Addgene) were gifts from Xingxu Huang (ShanghaiTech University). The pre-gRNA vector was linearized by *BspQ*I (New England Biolabs, Beverly, MA, USA) digestion. The targeted gRNA oligos were synthesized and inserted into the linearized pre-gRNA plasmid by T4 ligase (New England Biolabs). The sequences of all the gRNAs used in this study are listed in Supplementary Table 1 (see section on supplementary materials given at the end of this article). U6-pre-gRNA sequence was cloned from a pre-gRNA plasmid and further inserted into a Cas13d-expressing plasmid to construct the binary expression system, named Cas13d-gRNA. The construction of a knockin vector for site integration in the porcine genome has been previously described (Zheng *et al.* 2017). Briefly, the UCP1-gRNA protospacer sequence was cloned from porcine genome and further inserted into a Cas13d-gRNA vector to complete the donor vector, named UCP1-U6-pre-gRNA-CAG-Cas13d-GFP-UCP1. The UCP1-targeted CRISPR/Cas9 vector (Cas9-UCP1-gRNA) was obtained as previously described (Zheng *et al.* 2017).

### Cell culture and transfection

Porcine embryo fibroblast (PEF) cells were isolated from 35-day-old porcine fetuses as previously described (Huang *et al.* 2016). PEF cells were co-transfected with Cas13d and pre-gRNA plasmids (or Cas13d plasmid only as a control) by nucleofection with a 2B Nucleofector^TM^ device (Lonza, Basel, Switzerland) using the program (U-023). After 48 h, transfected cells were subjected to FACS sorting based on the expression of GFP that co-expressed with Cas13d and were collected for subsequent analysis. For single colony screening, the UCP1-U6-pre-gRNA-CAG-Cas13d-GFP-UCP1 and Cas9-UCP1-gRNA vectors were co-transfected via nucleofection, similarly. Single cells were plated into each well of 96-well plates by FACS and cultured for about 10 days to form colonies. The cell colonies were genotyped by PCR using 2× Taq Master Mix (Vazyme, Nanjing, Jiangsu, China) followed by Sanger sequencing.

### Gene expression analysis

Total RNA was extracted from GFP-positive cells using TRIzol reagent (Invitrogen). One microgram of total RNA was then reverse transcribed using TB Green® Premix Ex Taq™ (Tli RNaseH Plus) Kit (TaKaRa) according to the manufacturer’s instructions and used as a template for subsequent quantitative PCR to evaluate the expression of target genes at the mRNA level. The housekeeping gene, GAPDH or H2AFZ, was used as the internal control. The reaction was performed at 95°C for 1 min followed by 40 cycles of 95°C for 10 s and 60°C for 40 s by using a 2×SYBR Green Mix (TaKaRa) on a BIO-RAD CFX 96 system. The sequences of the primers are shown in Supplementary Table 2. Relative gene expression was calculated by the comparative CT method (2^−ΔΔCt^). For semi-quantitative PCR, two-cell embryos were collected to extract total RNA using a Cells-to-CT 1-Step Power SYBR Green Kit (Thermo Fisher Scientific). The sequences of primers are listed in Supplementary Table 2.

### Western blot

Cells were lysed at 48 h post-transfection with BROD (Thermo Fisher Scientific) buffer for 30 min on ice, then denatured with loading buffer boiled at 100°C for 10 min. Protein samples were separated by SDS-PAGE gel electrophoresis and transferred onto a PVDF membrane. Membranes were blocked with 5% skim milk at 37°C for 1 h, then incubated with primary antibodies at 4°C overnight. Primary antibodies included KDM5B (A7772, 1:1000, ABclonal, Wuhan, Hubei, China) and GAPDH (CW0100M, 1:5000, CWBIO, Nanjing, Jiangsu, China). After rinsing three times in TBST, the membranes were incubated at 37°C for 1 h with HRP-conjugated secondary antibodies (BE0101-100 or BE0102-100 Easybio, Beijing, China). After rinsing three times in TBST, membranes were treated with super ECL Plus Western Blotting Substrate (Thermo Fisher Scientific) and signals were visualized using a Tanon 5200 (Tanon Science & Technology Co. Ltd, Shanghai, China).

### Cell proliferation and cell cycle analysis

At 24 h after transfection, GFP-positive cells were subjected to FACS sorting and collected in 96-well plates at a density of 5000 cells per well. Cell proliferation was assayed using a Cell Counting Kit-8 kit (Dojindo, Kumamoto, Japan) at 0, 24, 48, 72, and 96 h. For cell cycle analysis, the GFP-positive cells were collected at 72 h after transfection and stained with Hoechst 33342 at 37°C for 15 min. Further, FACSCalibur flow cytometer and FlowJo cell cycle were used to analyze the percentage of cells in each phase.

### Oocyte microinjection

Porcine ovaries were collected from a local slaughterhouse and transported to the laboratory in 0.9% saline maintained at 37°C. The methods of oocyte collection, *in vitro* maturation, and microinjection, were described previously (Yao *et al.* 2014). Cas13d mRNA and gRNA targeting *KDM5B* were* in vitro* transcribed according to the protocol of Message Machine T7 Ultra Kit (Thermo Fisher Scientific) and MEGAshortscript Kit (Thermo Fisher Scientific), respectively. They were injected into the cytoplasm of matured MII stage oocytes using a FemtoJet microinjector (Eppendorf, Hamburg, Germany) as previously described (Huang *et al.* 2015). Injected oocytes were activated by electric pulse via ECM 2001 (BTX, Holliston, MA, USA) according to the manufacturer’s instructions and then cultured at 39°C in 5% CO_2_ in porcine zygote medium 3 (PZM3).

### Immunofluorescence staining

Cells or embryos at the indicated time points were fixed in 4% paraformaldehyde at room temperature for 30 min after washing in PBS three times, then permeabilized in 0.05% Triton X-100 in PBS for 20 min at room temperature and blocked at 37°C for 1 h in 1% BSA. The samples were incubated with primary antibodies against H3K4me3 (ab8580, 1:1000; Abcam), H3K9me3 (ab8898, 1:500, Abcam), H3K27me3 (A16199, 1:100, ABclonal), H3K4ac (07-539, 1:1000, Millipore), H4K8ac (07-328, 1:1000, Millipore), and H4K12ac (07-595, 1:1000, Millipore) overnight at 4°C, according to the manufacturer’s instructions. The samples were then incubated with secondary antibodies (ZF-0316, 1:200, ZSBIO, Beijing, China) after washing with PBS for 1 h at 37°C. After washing three times, samples were mounted on slides using Prolong gold antifade reagent with DAPI (Thermo Fisher Scientific). Images were obtained using a ZEISS 780 system. Fluorescence intensity was measured with IPP software, and all images were assembled without any adjustment.

### Statistical analysis

All the experiments were repeated at least three times, and each experiment was performed in triplicate. All statistical analysis was performed with GraphPad prime software. Student’s two-tailed t-test was used to analyze experiments involving only two groups, and one-way ANOVA was used to analyze data involving three or more groups. Results were presented as mean ± s.e.m. Values of *P* < 0.05 were considered statistically significant.

## Results

### Cas13d mediated RNA knockdown in porcine fibroblast cells

Cas13d has been reported to be capable of processing CRISPR arrays (pre-gRNA) to mature gRNA in HEK293T (293T) cells, and that gRNA could target various genes including *NF2* and *STAT3* (Konermann *et al.* 2018). To assess whether CRISPR/Cas13d could knockdown target RNAs in porcine cells, we first designed two homologous gRNAs targeting porcine *NF2* and *STAT3* as reported in 293T cells (Supplementary Table 1). Target sites with highly similar sequences in pig and human *NF2* and *STAT3* genes were selected to design gRNA. We confirmed the knockdown efficiency of *NF2* and *STAT3* mediated by CRISPR/Cas13d in 293T cells, which reached about 70% (Fig. 1B and C). Similarly, *NF2* and *STAT3* mRNA were significantly decreased in Cas13d and gRNA transfected PEF cells, and the efficiency was about 50% (Fig. 1B and C). These results showed that CRISPR/Cas13d could target mRNAs in porcine cells efficiently.

To further confirm the feasibility of CRISPR/Cas13d mediated mRNA knockdown in pigs, *KDM5B* (lysine-specific histone demethylase 5B) was selected as a target gene. KDM5B is an H3K4me3 demethylase that catalyzes the demethylation of histone H3K4. Four gRNAs were designed to target *KDM5B* both in 293T cells and PEFs, one of the gRNA (gRNA4) sequences is shown in Fig. 1D. In 293T cells, all four gRNAs could mediate *KDM5B* knockdown efficiently, and gRNA4 exhibited the highest efficiency (98%). In PEF cells, the expression of *KDM5B* was downregulated to 70 and 50% by gRNA3 and gRNA4, respectively (Fig. 1E). The gRNA4 with the highest cleavage efficiency was chosen to target *KDM5B* for the following assays. Then, the optimal time point for detecting the knockdown of *KDM5B* upon transfection was determined by a time-course analysis. Cas13d expression vector and gRNA4 were co-transfected into PEF cells, and the expression level of *KDM5B* was detected at 12, 24, 48, 72, and 96 h post-transfection through quantitative PCR after FACS. Results showed that the relative expression of *KDM5B* mRNA was decreased by 70% 24 h post-transfection, while the knockdown efficiency declined in later time points (Fig. 1F). Western blot analysis further confirmed the downregulation of *KDM5B* by the Cas13d system at the protein level (Fig. 1G).

### The effects of *KDM5B* knockdown to epigenetic regulation

**Figure 2:**
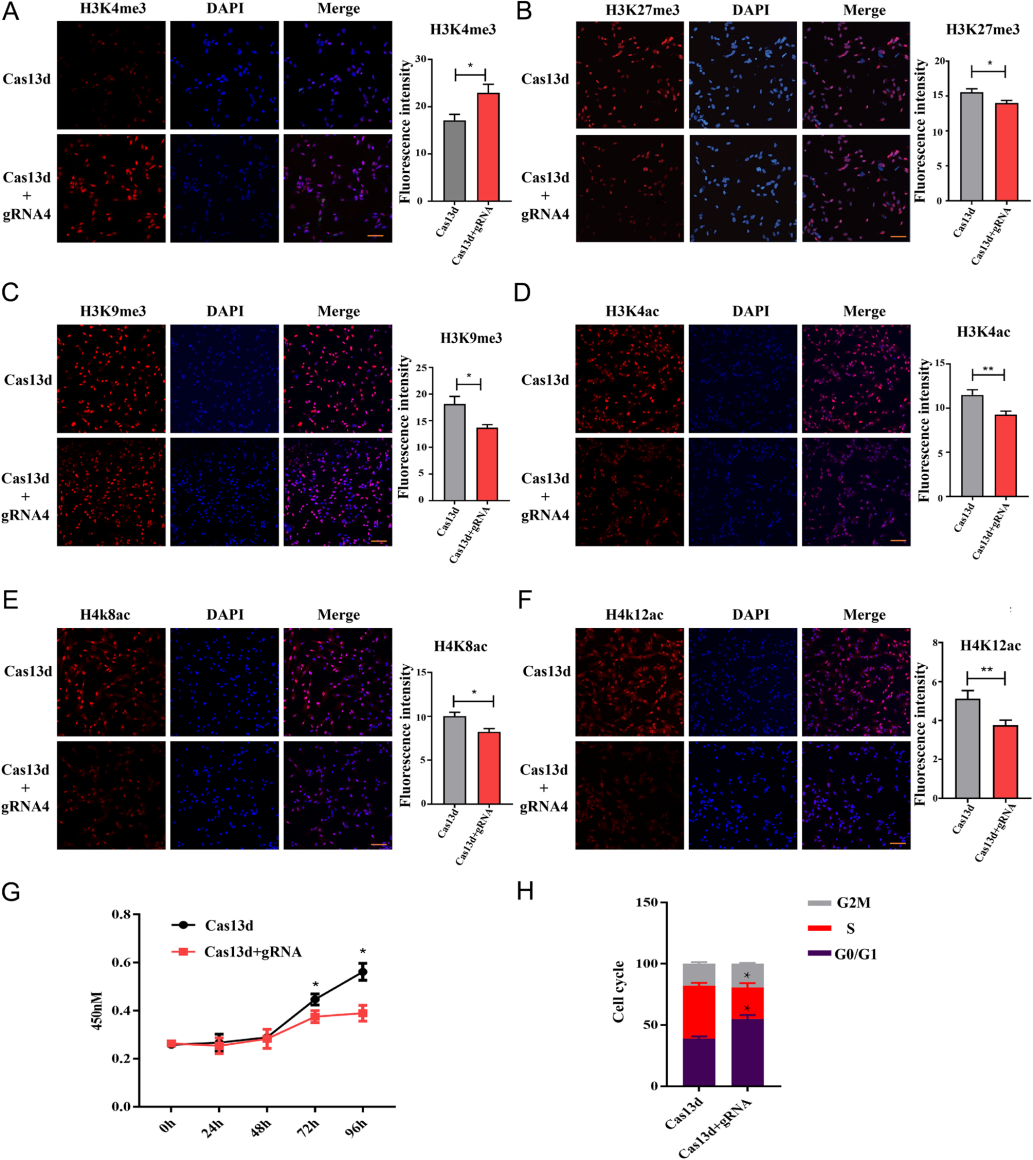
Knockdown of KDM5B resulted in the abundance change of histone modifications and the arrest of PEF cell proliferation and cell cycle. (A, B, C, D, E and F) Immunofluorescence staining of H3K4me3 (A), H3K27me3 (B), H3K9me3 (C), H3K4ac (D), H4K8ac (E), and H4K12ac (F)in PEF cells transfected with Cas13d and gRNA4 targeting KDM5B or Cas13d only. The quantitative analysis of fluorescence intensity is shown on the right. Data are presented as the mean ± s.e.m.; *P < 0.05. Scale bar, 40 μm. At least 10 randomly selected fields were counted for each group. (G) Cell proliferation analysis of PEF cells transfected with Cas13d and gRNA4 (Cas13d only as control). *P < 0.05. 450 nm, the optical density (OD) absorbance at the wavelength of 450nm. (H) Cell cycle analysis of PEF cells transfected with Cas13d and gRNA4 (Cas13d only as control.) *P < 0.05.

To examine the influence of *KDM5B* knockdown on histone methylation in PEF cells, H3K4me3 levels were analyzed by immunofluorescence staining. Results revealed that the knockdown of *KDM5B* significantly increased H3K4me3 levels (Fig. 2A). Observation of enhanced H3K4me3 expression upon *KDM5B* knockdown, as well as a previously reported bivalent balance between H3K4me3 and H3K27me, encouraged us to examine the abundance of H3K27me3 (Huang *et al.* 2015, Liu *et al.* 2016). Immunofluorescence results reproduced the findings that knockdown of *KDM5B* increased the abundance of H3K4me3 and decreased the abundance of H3K27me3 (Fig. 2B). In parallel, a lower level of H3K9me3 was observed in the knockdown group (Fig. 2C). Given the interaction between histone methylation and acetylation (Nicolas *et al.* 2003), and the interaction between KDM5B and HDAC (Ma *et al.* 2012, Wang *et al.* 2016), we examined some histone acetylation markers’ response to the knockdown of *KDM5B*. Results showed that the fluorescent signal of H3K4ac, H4K8ac, and H4K12ac decreased significantly in the *KDM5B*-knockdown cells (Fig. 2D, E and F). Next, the proliferation of the Cas13d-cleaved cells was examined, and results showed the proliferation rate of the *KDM5B*-knockdown cells was much lower than that of the control group (Fig. 2G). To determine whether the impaired cell proliferation was due to cell cycle arrest, the cell cycle was checked 72 h post-transfection with Cas13d and gRNA. As expected, the inhibition of *KDM5B* expression caused many PEF cells to arrest at the G0/G1 phase and fail to enter the S phase (Fig. 2H). The above results demonstrated that Cas13d-mediated *KDM5B* knockdown led to changes in histone methylation and acetylation modifications, cell proliferation retardation, and cell cycle arrest in PEF cells.

### Cas13d-mediated continuous knockdown of *KDM5B*

**Figure 3:**
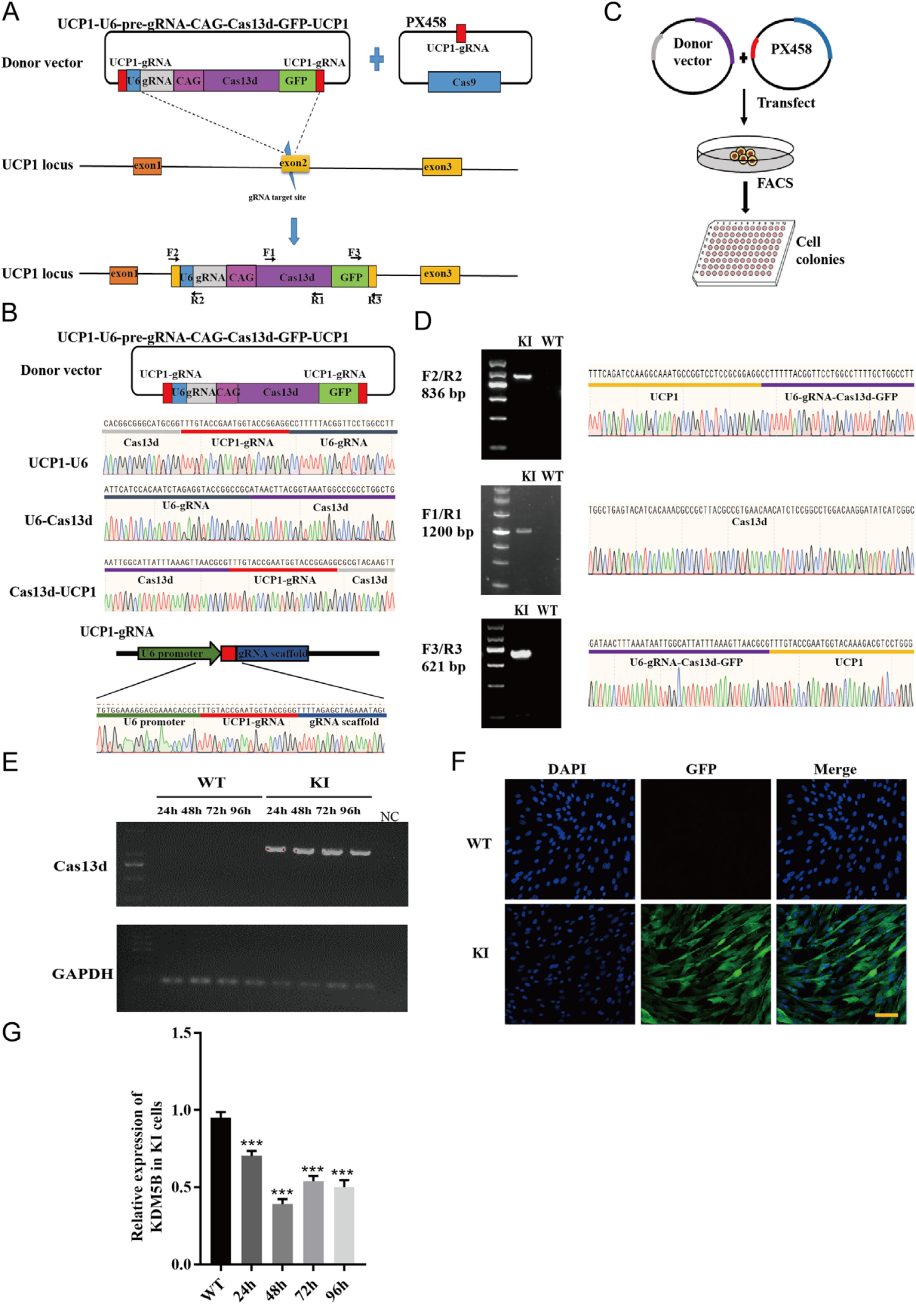
Cas13d-mediated continuous knockdown of KDM5B in PEF cells. (A) Schematic representation of the design of CRISPR/Cas9-mediated, HR-independent integration of Cas13d and gRNA. Exons of UCP1 are shown in yellow rectangle. The blue lightning bolt in exon2 represents the Cas9-UCP1-gRNA target site, and the red boxes represent UCP1-gRNA. The U6-pre-gRNA-CAG-Cas13d-GFP expression plasmid is shown on the upper left, and the Cas9-UCP1-gRNA expression plasmid is shown on the upper right. The bottom showed forward integration of Cas13d and gRNA in UCP1. Three pairs of primers were designed for genotyping. F2/R2 and F3/R3 were designed for genotyping the 5’ and 3’ junctions in the transgenic colonies, respectively. F1/R1 were designed for detecting the integration of the UCP1-U6-pre-gRNA-CAG-Cas13d-GFP-UCP1 expression plasmid. (B) Confirmation of the successful construction of UCP1-U6-pre-gRNA-CAG-Cas13d-GFP-UCP1 and Cas9-UCP1-gRNA vector by Sanger sequencing. (C) Schematic diagram of the process to generate Cas13d-gRNA knockin cell colonies. (D) Genotyping results of the positive knockin colony by PCR (left) and Sanger sequencing (right). The primers used for PCR is indicated in A. (E) Semi-quantitative PCR analysis of the expression of Cas13d from the WT and KI cell at different time points post-transfection. (F) The GFP expression in the positive KI colony was detected by immunofluorescence imaging. Scale bar, 40 μm. (G) Quantitative PCR analysis of the expression of KDM5B in the KI colony against WT in different times.

In order to explore the possibility of downregulating the target gene persistently, a CRISPR/Cas9-mediated, HR-independent integration strategy was used to insert Cas13d and *KDM5B*-gRNA4 into the porcine endogenous* UCP1* locus (Fig. 3A). *UCP1* is reported to be a pseudogene, which can be used as a safe harbor site to insert exogenous genes (Zheng *et al.* 2017). The UCP1-U6-pre-gRNA-CAG-Cas13d-GFP-UCP1 containing bait sequence (the same sequence as the Cas9 target site in the genomic locus) was constructed as previously described (Zheng *et al.* 2017), with Cas13d driven by the CAG promoter and gRNA driven by the U6 promoter. A Cas9-UCP1-gRNA plasmid targeting the endogenous *UCP1* was also constructed (Fig. 3B). The Cas9-UCP1-gRNA plasmid and UCP1-U6-pre-gRNA-CAG-Cas13d-GFP-UCP1 were co-transfected into PEF cells, and cells were seeded into 96-well plates by FACS to screen single colonies (Fig. 3C). Cell colonies were propagated and genotyped using primers designed to identify forward integration (F2/R2 for 5′ junction, F1/R1 for Cas13d detection, F3/R3 for 3′ junction). One colony was identified as positive for forward integration (Fig. 3D). Furthermore, Cas13d expression was confirmed by semi-quantitative PCR in this colony (Fig. 3E). To evaluate the expression of Cas13d, GFP was fused with Cas13d driven by a CAG promoter in the targeting vector. Results showed that the fused GFP was express consistently, which indicated the successful expression of Cas13d (Fig. 3F). Quantitative PCR results showed that the expression of *KDM5B* was continuously impeded at the mRNA level (Fig. 3G) in this colony. These results suggested that site-specific Cas13d-knockin could mediate *KDM5B* knockdown continuously.

### Cas13d-mediated *KDM5B* knockdown in porcine parthenogenetic embryos by direct microinjection

**Figure 4:**
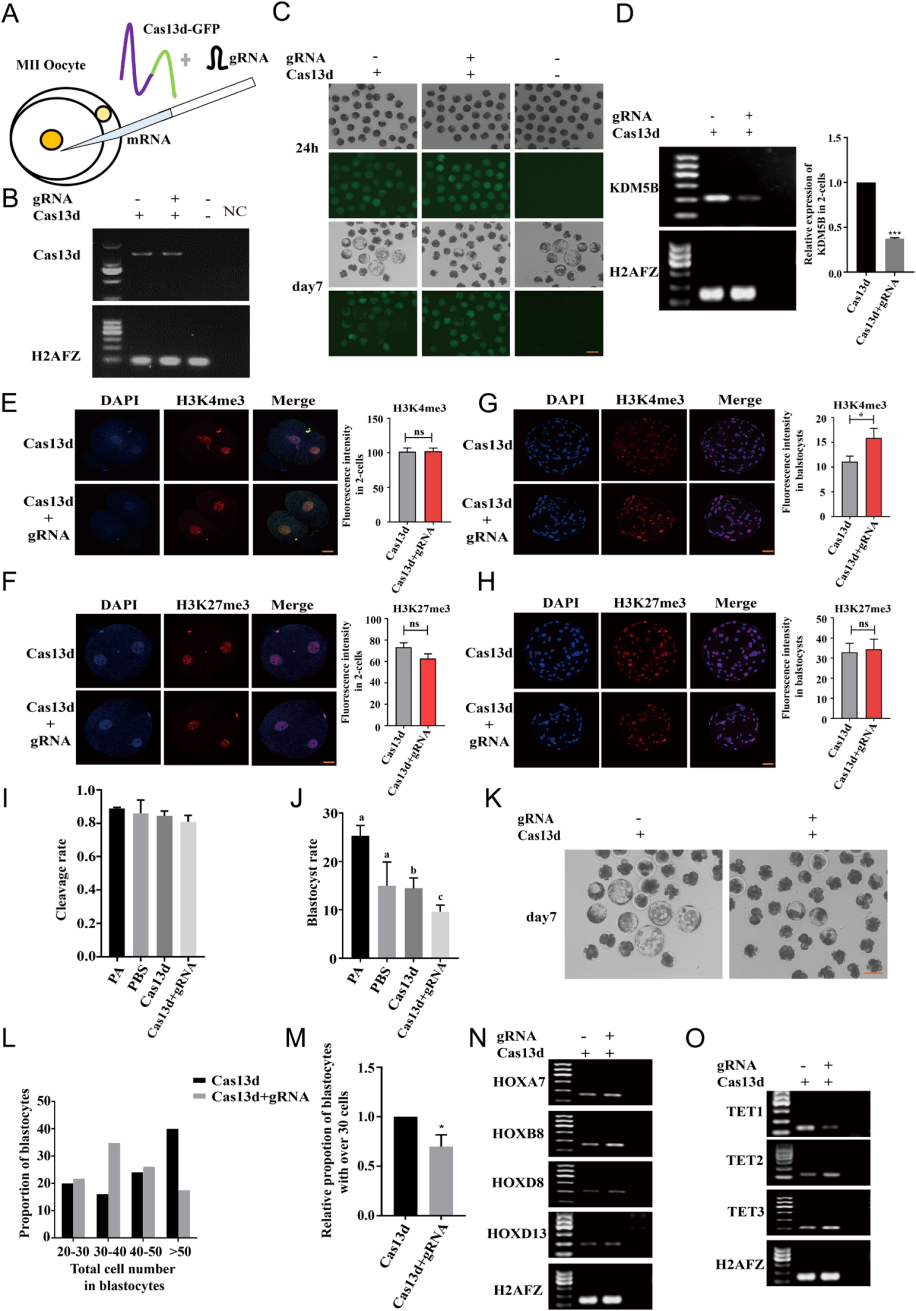
Cas13d-mediated KDM5B knockdown in porcine oocytes. (A) Schematic diagram of microinjection in MII oocytes. (B) Expression of Cas13d transcript in blastocysts after injection of Cas13d mRNA with or without gRNA was determined by semi-quantitative PCR. (C) GFP expression in two-cell embryos and blastocysts after injection of Cas13d mRNA with or without gRNA. Scale bar, 40 μm. (D) Semi-quantitative PCR analysis of expression of KDM5B after injection Cas13d with or without gRNA in two-cell embryos is shown on the left. The quantitative result is shown on the right. (E, F, G and H) Immunofluorescence staining of H3K4me3 and H3K27me3 in two-cell embryos (E and F) and blastocysts (G and H) after injection of Cas13d RNA with or without gRNA. The quantitative results are shown on the right. At least 10 embryos were counted for each group. Scale bar, 40 μm. (I) The cleavage rate in both groups with or without injection. (J and K) The blastocyst rate in both groups with or without injection. A representative picture of embryos developed to the blastocyst stage in both groups is shown in K. (L) Proportion of blastocysts with different cell number was counted after injection of Cas13d with or without gRNA. (M) The proportion of blastocysts with over 30 cells was compared after injection Cas13d and gRNA vs Cas13d only. (N and O) Semi-quantitative PCR analysis of the expression of HOX genes and TET genes in two-cell embryos.

To confirm whether Cas13d can target *KDM5B* in oocytes effectively, Cas13d mRNA and gRNA were microinjected into porcine MII oocytes, and the knockdown efficiency, expression of epigenetic markers, and embryo developmental competence were examined. First, Cas13d mRNA and gRNA4 against the *KDM5B*-coding sequence were transcribed *in vitro*. To indicate the expression of Cas13d, GFP was co-expressed with Cas13d by the single CAG promoter. Next, Cas13d mRNA and gRNA were microinjected into MII oocytes, and the oocytes were parthenogenetically activated (Fig. 4A). Distinct GFP expression was observed in the Cas13d injection group in blastocysts, and Cas13d expression was confirmed by semi-quantitative PCR of blastocysts (day 7), which suggested the expression of Cas13d mRNA could continue to the blastocyst stage (Fig. 4B and C). The transcription level of *KDM5B* was then detected by semi-quantitative PCR, which showed significant downregulation in two-cell embryos that underwent Cas13d and *KDM5B*-gRNA injection (Fig. 4D). Immunofluorescence staining of H3K4me3 and H3K27me3 revealed no differences between the two-cell embryo knockdown and control groups, while the abundance of H3K4me3 in blastocysts increased significantly by *KDM5B* knockdown (Fig. 4E, F, G and H). In addition, no differences were observed in the cleavage rate between the *KDM5B*-knockdown and the control group (Fig. 4I). However, in the *KDM5B*-knockdown group, the developmental competence of parthenogenetic embryos reaching the blastocyst stage was significantly reduced (Fig. 4J and K), and the total cell number of the blastocysts were significantly decreased (Fig. 4L and M). Previous findings had reported the disturbing bivalent balance of H3K4me3 and H3K27me3 activated the expression of *HOX* genes (Gao *et al.* 2010, Huang *et al.* 2015, Zheng *et al.* 2016), which our study recapitulated. Our semi-quantitative PCR results showed that the expression of multiple *HOX* family members (*HOXA7*, *HOXB7*, *HOXD8*, and *HOXD13*) was greatly elevated (Fig. 4N). Further, ten-eleven translocation (TET) gene family members had been reported to regulate interactions between DNA methylation and histone modification in mouse embryonic stem cells (ESCs), and our results showed that *TET2* and *TET3* were increased and *TET1* was decreased by knockdown of *KDM5B*, which might be a reason for the decreased developmental competence (Fig. 4O). Thus, our findings suggest that CRISPR/Cas13d could be used to target endogenous genes in porcine early embryos and facilitate the study of their function during embryonic development.

## Discussion

Recently, a novel RNA targeting technology, CRISPR/Cas13, was developed and reported to be able to bind and cleave targeting RNA (Ali *et al.* 2018). Compared to Cas13a/b/c, Cas13d protein is approximately 20% smaller, only 930aa, making it easier to package into the adeno-associated virus (AAV) for primary cell transfection and *in vivo* delivery. Furthermore, RNA target cleavage of Cas13d did not appear to depend on the PFS. Cas13d could achieve higher knockdown efficiency relative to other members of the Cas13 family (Granados-Riveron & Aquino-Jarquin 2018, Konermann *et al.* 2018). CRISPR/Cas13d, shRNA, and CRISPRi were used to target three endogenous transcripts, including *B4GALNT1*, *ANXA4*, and *HOTTIP*, and provided an average knockdown of 96% for Cas13d, 65% for shRNA, and 53% for CRISPRi (Konermann *et al.* 2018), suggesting CRISPR/Cas13d provides a more efficient strategy for gene expression manipulation. Although RNAi has been a prominent tool for RNA knockdown, gene function analysis, and pathway analysis, off-target effects are still the greatest challenges for RNAi application (Patzel *et al.* 2005). The specificity of RNAi is dependent on RNA target recognition by siRNA, while the short length (~22 bp) of siRNA limits sequence specificity. Additional problems can occur if siRNA is targeting RNA with a high tolerance for mismatches. Indeed, widespread off-target effects of RNAi were detected and difficult to diminish (Jackson *et al.* 2003). Apart from the complementarity between gRNA and target RNA, CRISPR/Cas13 families show improved specificity with identifiable PFS sequences. Even though a PFS preference of Cas13d has not yet been found, no off-target effects were detected. The activity of CRISPR/Cas13d is severely decreased or eliminated if 1–2 mismatches occur in the seed region of gRNA, which greatly reduces the risk of off-target effects. Konermann *et al*. utilized Cas13d and shRNA to target *B4GALNT1* and demonstrated widespread off-target transcriptional changes with shRNA but no significant off-target changes for Cas13d (Abudayyeh *et al.* 2017, Konermann *et al.* 2018). Considering the high specificity and targeting efficiency of Cas13d, we aimed to build a platform of Cas13d-mediated mRNA knockdown in porcine cells and early embryos to facilitate future application in pig research. We first confirmed the knockdown effects of *NF2* and *STAT3* genes in 293T cells and PEFs. An obvious knockdown of *NF2* and *STAT3* in porcine fibroblast cells was achieved. Further, CRISPR/Cas13d-mediated *KDM5B* knockdown was also observed in 293T and PEF cells. Interestingly, as Cas13 may efficiently cleave single-strand regions near attachable target RNA rather than within a higher-order RNA structure, different gRNAs displayed various knockdown efficiency (Abudayyeh *et al.* 2016). Previous research has shown that CRISPR/Cas13a-mediated cleavage occurred at uracil residues (Abudayyeh *et al.* 2016, Smargon *et al.* 2017). Uracil incorporation in target RNA differs in number and location, which may be another reason for the diverse knockdown efficiency of various gRNAs. Studies have shown that the knockdown effect of CRISPR/Cas13d in 293T cells is reversible (Cox *et al.* 2017), and our experiments showed that knockdown efficiency was greatest at 24 h post-transfection and decreased over time, which is beneficial for gene therapy in RNA level in a reversible and instantaneous way. Ultimately, CRISPR/Cas13d enables robust RNA cleavage, making it a promising candidate for highly efficient RNA knockdown in pigs.

KDM5B is a member of the JARID1/KDM5B family, which has been shown to demethylate H3K4me3, a chromatin marker associated with transcriptional activity (Kidder *et al.* 2014). Bivalent modifications of H3K4me3 and H3K27me3 at the same location have been demonstrated to play a pivotal role in pluripotency in ESCs (Azuara *et al.* 2006, Bernstein *et al.* 2006, Gan *et al.* 2007). Our results showed that the downregulated expression of *KDM5B* using Cas13d in PEF cells resulted in a dramatic increase in H3K4me3 levels and a decrease in H3K27me3 levels (Mansour *et al.* 2012, Chen *et al.* 2013, Matoba *et al.* 2014, Xie *et al.* 2016, Jullien *et al.* 2017). Meanwhile, the abundance of the repressive marker, H3K9me3, decreased after the knockdown of *KDM5B*. There was cross-talk between histone methylation and acetylation. For example, Ikaros-mediated repression of *KDM5B* depends on binding histone deacetylase (HDAC) to the upstream regulatory elements of KDM5B, regulating the epigenetic signature in leukemia (Wang *et al.* 2016). HDAC1 and HDAC2 were previously reported to regulate H3K4 methylation through KDM5B (Ma *et al.* 2012). Thus, considering the possible interaction between HDAC and KDM5B, we examined whether histone acetylation modifications, including H3K4ac and H4K8/12ac, were decreased by *KDM5B* knockdown. Changes in histone modifications may result in changes in the expression levels of related genes and further affect cell proliferation and cell cycle. Several studies showed that KDM5B played an important role in cell proliferation. Knockdown of *KDM5B* notably inhibited hepatocellular carcinoma (HCC) cell proliferation through arresting the cell cycle at G1/S (Scibetta *et al.* 2007, Schmitz *et al.* 2011). Similarly, our results demonstrated that knockdown of *KDM5B* mediated by CRISPR/Cas13d led to changes of histone methylation and acetylation modifications, resulting in the arrest of cell proliferation and cell cycle in PEF cells. Together, our results suggest that CRISPR/Cas13d provides a feasible and effective method for gene function research in pigs.

Next, to overcome the transient knockdown effects of Cas13d, we exploited CRISPR/Cas9 technology to achieve long-term knockdown of *KDM5B* in PEFs. CRISPR/Cas9 mediated site-specific insertions of exogenous DNA sequences have been successfully reported (Li *et al.* 2015, Zheng *et al.* 2017). In our study, we inserted a 7 kb U6-gRNA-CAG-Cas13d-GFP fragment into the porcine endogenous *UCP1* locus using a CRISPR/Cas9-mediated, HR-independent approach. The stable integration of Cas13d and gRNA resulted in the continuous expression of Cas13d and steady knockdown of *KDM5B*. Swine RNA viruses, such as classical swine fever virus (CSFV), porcine reproductive and respiratory syndrome virus (PRRSV), and foot-and-mouth disease virus (FMDV), remain a great threat to animal health and the pig industry. Anti-CSFV pigs were previously produced by inserting antiviral shRNAs at the porcine Rosa26 locus via a CRISPR/Cas9-mediated knockin strategy, and these pigs could effectively limit replication of CSFV to protect the pigs from Swine Fever (Li *et al.* 2011). In addition, a report has indicated that the CRISPR/Cas13b system could effectively knock down PRRSV genomic RNA and sub-genomic RNAs (Cui *et al.* 2020). Our results here showed that CRISPR/Cas13d could successfully integrate into the porcine UCP1 locus (a safe harbor site) and therefore maintain continuous cleavage of target RNAs, which will provide a basis for creating anti-virus pigs in the future.

Active removal of broad H3K4me3 domains by demethylases, such as KDM5B, is essential for early embryo development. It has been found that knockdown of *KDM5B* impaired blastocyst formation on 3.5 and 4.5 days (Liu *et al.* 2016), and knockout of *KDM5B* caused early embryonic lethality (Gao *et al.* 2010, Catchpole *et al.* 2011, Albert *et al.* 2013). The *KDM5B* gene is most active in mouse 2-cell embryos, while the expression pattern of this gene was very different in pigs. In pigs, *KDM5B* expression was very low in porcine two-cell embryos, increased in four-cell embryos, and reached a peak at the blastocyst stage (Huang *et al.* 2015). In mice, most (87%) of the *KDM5B*-depleted embryos failed to develop to the blastocyst stage (Dahl *et al.* 2016). Our results showed that knockdown of *KDM5B* expression impairs porcine preimplantation embryo development. *KDM5B* has been shown to occupy promoter regions of several *HOX* genes and regulate *HOX* gene activity (Agger *et al.* 2007, VerMilyea *et al.* 2009). The expression of several *HOX* family members (*HOXA*, *HOXB7*, *HOXD8*, and *HOXD13*) also increased in *KDM5B* downregulated porcine embryos. And knockdown of *KDM5B* increased the H3K4me3 abundance at the blastocyst stage. All our data showed that CRISPR/Cas13d could downregulate the transcription level of histone modification-related genes in porcine embryo to regulate their histone modifications, and further regulate embryos development. So CRISPR/Cas13d can potentially be used as a potent tool to interfere with the expression of genes that playing a crucial role during embryo development to regulate early embryo development.

In summary, CRISPR/Cas13d-mediated endogenous gene knockdown at the transcription level was accomplished in porcine fetal fibroblasts cells transiently, as well as permanently, and in porcine parthenogenetic embryos, which will provide a novel tool for gene regulation and programmable RNA editing.

## Supplementary Material

Table S1. gRNAs used in this study

Table S2. primers used in this study

## Declaration of interest

The authors declare that there is no conflict of interest that could be perceived as prejudicing the impartiality of the research reported.

## Funding

This work was supported by the China National Key R&D Program (2020YFC1316600 and 2020YFA0509503), the National Natural Science Foundation of China (31925036, 81671274, 31801031, 31601008, 31701073 and 32025034), the Strategic Priority Research Programs of CAS (XDA16030101), and the National Transgenic Project of China (2016ZX08009003-006-007).

## Author contribution statement

All authors listed have made a substantial, direct and intellectual contribution to the work, and approved it for publication.
