# Supplementary material for: CRISPR/Cas13d-mediated efficient KDM5B mRNA knockdown in porcine somatic cells and parthenogenetic embryos
Source: Reproduction. 2021 Jun 7;162(2):149–60. doi: 10.1530/REP-21-0053 (PMC8284906; doi:10.1530/REP-21-0053)
Supplement: Table S1. gRNAs used in this study [file supplementary_table_1.pdf]

Supplementary materials

Table S1. gRNAs used in this study

Site	gRNA sequence
NF2-human-gRNA-1	CTTGGCCTGGACGGCGTAAGAAGCCAGGAG
NF2-pig -gRNA-1	CTTGGCCTGCACGGCGTAAGAAGCCAGGAG
NF2-human-gRNA-2	CTTGTGAACACTGGGGTCGTAGTCACCATA
NF2-pig -gRNA-2	CTTGTGAACAGAGGGGTCGTAGTCGCCGTA
STAT3-human-gRNA-1	ATCACAATTGGCTCGGCCCCCATTCCCACA
STAT3-pig-gRNA-1	GTCGCAATTGGCTCGGCCCCCGTTCCCACA
STAT3-human-gRNA-2	ATCAGGGAAGCATCACAATTGGCTCGGCCC
STAT3-pig-gRNA-2	ATGAGGGAGGCGTCGCAATTGGCTCGGCCC
KDM5B-human-gRNA-1	CTTGGCCTGGACGGCGTAAGAAGCCAGGAG
KDM5B-pig-gRNA-1	CTTGGCCTGCACGGCGTAAGAAGCCAGGAG
KDM5B-human-gRNA-2	CTTGTGAACACTGGGGTCGTAGTCACCATA
KDM5B-pig-gRNA-2	CTTGTGAACAGAGGGGTCGTAGTCGCCGTA
KDM5B-human-gRNA-3	ATCACAATTGGCTCGGCCCCCATTCCCACA
KDM5B-pig-gRNA-3	GTCGCAATTGGCTCGGCCCCCGTTCCCACA
KDM5B-human-gRNA-4	ATCAGGGAAGCATCACAATTGGCTCGGCCC
KDM5B-pig-gRNA-4	ATGAGGGAGGCGTCGCAATTGGCTCGGCCC
