# Supplementary material for: CRISPR/Cas13d-mediated efficient KDM5B mRNA knockdown in porcine somatic cells and parthenogenetic embryos
Source: Reproduction. 2021 Jun 7;162(2):149–60. doi: 10.1530/REP-21-0053 (PMC8284906; doi:10.1530/REP-21-0053)
Supplement: Table S2. primers used in this study [file supplementary_table_2.pdf]

Supplementary materials

Table S2. primers used in this study

Primer name	Sequence	Product size (bp)
NF2-human-F	GCTGGTTCAGGAGATCACACA	193bp
NF2-human-R	CCTTTTTGGAAGCAATTCCTCTTG	
NF2-pig-F	GCTGGTTCAGGAGATCACGCA	193bp
NF2-pig -R	CCTTTTTGGAAGCAGTTCCTCTTG	
STAT3-human-F	AAACACTTGACCCTGAGGGAG	125bp
STAT3-human-R	AGGCCTTGGTGATACACCTC	
STAT3-pig-F	AAACACCTGACCCTGAGAGAG	125bp
STAT3-pig-R	AGGCCTTGATGATACACCTC	
KDM5B-human-F	ATTGGAGGCCCAAACTCGTG	200bp
KDM5B-human-R	TCTTGGTAGCAATTTTGGTCC	
KDM5B-pig-F	ATTGGAGGCTCAAACTCGTG	200bp
KDM5B-pig-R	TCTTGGTGGCAATT TTGGTCC	
KI-F1	GGGCAACGTGCTGGTTATTG	12f00bp
KI-R1	CCACCTTCTCAGACACGGAC	
KI-F3	GCATCGATACCGTCGACCTC	622bp
KI-R3	CCTTTCCTGCCTTTCTGGCTT	
KI-F2	GTAGGGTGGAGTGGGGGTAAT	836bp
KI-R2	TATGCGGCCGGTACCTCTA	
H2AFZ-F	CCTCACCGCAGAGGTACTTG	146bp
H2AFZ-R	CACCACCACCAGCGATTGTA	
TET1-F	GGGATCTAAGCCTGCCAACA	132bP
TET1-R	AGGCTTCTGACTGGCAAAGG	
TET2-F	ATGCGTAGGTAAGTGCCAGG	123bp
TET2-R	CACAAATTGCTGCCAGACTCA	
TET3-F	GATTCACCCAGTGTCCAGGG	106bp
TET3-R	AGATTCGACATGGACCAGCG	
HOXA7-F	CTGCCGGACAACAAATCACA	151bp
HOXA7-R	CATAGCCGCTTCTCTGCGAG	
HOXB7-F	CTGCCGGACAACAAATCACA	151bp
HOXB7-R	CATAGCCGCTTCTCTGCGAG	
HOXD8-F	CGAAGTTTTACGGATACGAT	204bp
HOXD8-R	GTCTTCCTCTTCGTCTACCA	
HOXD13-F	CTTTCCAGGGGATGTGGCTC	180bp
HOXD13-R	CTCTCGGATAGGTTCGTGGC	
